# Loop-Mediated Isothermal Amplification Test for* Trypanosoma gambiense* Group 1 with Stem Primers: A Molecular Xenomonitoring Test for Sleeping Sickness

**DOI:** 10.1155/2017/8630708

**Published:** 2017-02-21

**Authors:** Zablon K. Njiru, Cecilia K. Mbae, Gitonga N. Mburugu

**Affiliations:** ^1^School of Health Sciences, Meru University of Science and Technology, P.O. Box 972-60200, Meru, Kenya; ^2^School of Health Professions, Murdoch University, Mandurah Campus, Education Drive, Mandurah, WA 6210, Australia; ^3^Kenya Medical Research Institute, Centre for Microbiology Research, P.O. Box 19464-00202, Nairobi, Kenya

## Abstract

The World Health Organization has targeted Human African Trypanosomiasis (HAT) for elimination by 2020 with zero incidence by 2030. To achieve and sustain this goal, accurate and easy-to-deploy diagnostic tests for Gambian trypanosomiasis which accounts for over 98% of reported cases will play a crucial role. Most needed will be tools for surveillance of pathogen in vectors (xenomonitoring) since population screening tests are readily available. The development of new tests is expensive and takes a long time while incremental improvement of existing technologies that have potential for xenomonitoring may offer a shorter pathway to tools for HAT surveillance. We have investigated the effect of including a second set of reaction accelerating primers (stem primers) to the standard* T. brucei gambiense* LAMP test format. The new test format was analyzed with and without outer primers. Amplification was carried out using Rotorgene 6000 and the portable ESE Quant amplification unit capable of real-time data output. The stem LAMP formats indicated shorter time to results (~8 min), were 10–100-fold more sensitive, and indicated higher diagnostic sensitivity and accuracy compared to the standard LAMP test. It was possible to confirm the predicted product using ESE melt curves demonstrating the potential of combining LAMP and real-time technologies as possible tool for HAT molecular xenomonitoring.

## 1. Introduction

The Human African Trypanosomiasis (HAT) is caused by two subspecies of trypanosomes, namely,* Trypanosoma brucei gambiense* which is prevalent in west and central Africa and causes chronic disease and* T. brucei rhodesiense* which occurs in East and southern Africa and causes acute form of disease. The parasites are transmitted through the bite of tsetse flies (*Glossina* spp.). The disease has been earmarked by the World Health Organization (WHO) for elimination by 2020 [1]. The goal being defined as having less than 1 case per 10 000 of the population in 90% of all foci [2] and with zero incidences by 2030 [3]. There is optimism that elimination will be achieved due to the current renewed control activities led by WHO and other partners. Indeed, the number of HAT cases reported had gone down to less than 5000 by 2014. In the 1960s the colonial health systems brought HAT under control but relaxation of the surveillance programmes led to resurgence of the disease [4], a mistake that should not be repeated today. Sustaining elimination and eventual eradication of HAT will require, among others, diagnostic tests that are accurate and easy to apply in endemic areas and that can be mass-produced at low cost to cover many samples since infection rates will go down. Of interest will be xenomonitoring tests that can be used in detection of pathogen in vectors and reservoir host as an alternative to screening populations. This is because compliance to screening may be reduced after HAT elimination. Therefore, monitoring of vectors for human infective trypanosomes will be an important assessment component in HAT elimination/eradication programs. The advantages of xenomonitoring are that the method is noninvasive and efficient in determining the presence of the pathogen in the target area. This approach has proven successful in the control of vector transmitted lymphatic filariasis [5].

The algorithms to diagnose Gambian HAT are often complex due to the unsatisfactory sensitivity and specificity of available tests. The methods include serological, parasitological, and those for staging the disease [6]. Recently, more serological tests with improved sensitivity and specificity have been developed, namely,* T. brucei gambiense*-specific SD Bioline HAT test [7, 8] and HAT sero-k-seT [9, 10], although further improvement is still required. Other recently launched tests include the* T. brucei* specific simple LED fluorescence microscope [11] and nucleic acid detecting RIME loop-mediated isothermal amplification (LAMP) test [12]. In addition, other experimental subgenus* Trypanozoon* LAMP tests have been reported [13–15] as well as more recently a* T. brucei gambiense* LAMP test [16]. It is postulated that the current serological tests will continue to play a dominant role in screening of HAT in population even after elimination while the LED microscopy and RIME LAMP test may have limited application in xenomonitoring for HAT due to their inability to exclude* T. b. brucei*. Therefore, there is need to develop tests that will specifically detect human pathogenic trypanosomes in vectors and in potential hosts.

Molecular characterisation of trypanosomes from Gambian HAT cases shows the vast majority of isolates belong to the genetically homogeneous group, Group 1* T. brucei gambiense* [17, 18], with Group 2* T. brucei gambiense* accounting for a smaller percentage [19]. Moreover, the* T. brucei gambiense*-specific glycoprotein (TgsGP) gene [20] was identified to be specific for Group 1 isolates and absent in Group 2 isolates [21]. Overall the percentage of Group 2 infection is so low and considered negligible and so far, only a few isolates exist in the laboratories. It is difficult to get a specific marker for Group 2 since the isolates are heterogeneous and genetically indistinguishable from* T. b. brucei*. PCR tests for* T. brucei gambiense* Group 1 based on TgsGP [22] and a* T. brucei rhodesiense* based on Serum Resistance Associated (SRA) gene [23] exist. These tests have low sensitivity limiting their application in HAT xenomonitoring.

It may be more advantageous in terms of cost and time to improve the existing HAT tests that show potential for xenomonitoring than developing new ones. One such test is the TgsGP* T. brucei gambiense* LAMP test [24]. Unfortunately, TgsGP is a low copy gene implying that even efficient amplification platform such as LAMP will only marginally improve sensitivity. Nevertheless, the specificity of TgsGP marker and the reported salient advantages of LAMP technology as a point of use platform make it desirable to investigate methods that may improve* T. brucei gambiense* LAMP test performance. One way is the inclusion of a second set of reaction accelerating primers called stem primers [25]. The stem primers anneal to the stem section of the sequence and sequentially amplify the target DNA with loop primers which anneal to the loop section of the target sequence [26]. This significantly increases the test product and hence increases the sensitivity. The use of stem primers was shown to improve sensitivity of HIV-LAMP test [25]; however no more follow-up studies have been done. In the present study, we have incorporated stem primers and significantly improved the detection of* T. brucei gambiense* DNA in tsetse fly samples. In addition, it was possible to confirm the predicted product through melt curves acquired in a portable real-time unit. Such integration of new technologies will contribute towards making LAMP technology a realistic surveillance tool for HAT.

## 2. Materials and Methods

### 2.1. Ethical Clearance

The samples from Uganda used in this study were collected from 1990 to 2005. The DNA were contributed as part a collaborative study for development of LAMP test for HAT at Murdoch University, Australia, as reported earlier [12]. The institutional Ethical Clearance for collection of human samples had been obtained from the Livestock Health Research Institute (LIRI), Tororo, Uganda, and the Uganda National Council of Science and Technology (UNCST), Kampala, Uganda, which records and regulates all research activities in the country. The use of samples from a HAT patient in Australia was approved by Royal Perth Hospital, Perth, Western Australia, through Dr. Christopher Heath as reported earlier [24]. All the samples were analyzed anonymously.

### 2.2. Sample Preparations

Two well characterised* T. brucei gambiense* DNA from isolates PT41 and B014 [12] initially isolated in Ivory Coast and Cameroon were used for analytical sensitivity and to spike tsetse fly samples, respectively. Tsetse midguts were prepared as described [27]. Briefly, tsetse fly midguts from nonexposed* G. pallidipes* were pooled in 5, 10, 15, and 20 and the whole dewinged fly into pools of 1, 5, and 10. Approximately ~10 pg (~100 trypanosomes/mL) of DNA from isolate B014 and double distilled water were added to each tube (Table 1). The samples were fully homogenized and divided into two portions. The first portion was processed with commercial DNA extraction kit (Qiagen, Victoria, Australia) and the resulting DNA was divided into aliquots and stored at −20°C. The second portion was mixed with an in-house prepared buffer composed of detergent and salts (0.1 M Tris-HCl, 0.05 M EDTA, and 1% SDS), boiled for 8 minutes, and centrifuged at 14,000 rpm for 4 min. The resulting supernatants were aspirated, divided into aliquots, and stored at −80°C.

### 2.3. LAMP Primers

**Figure 1:**
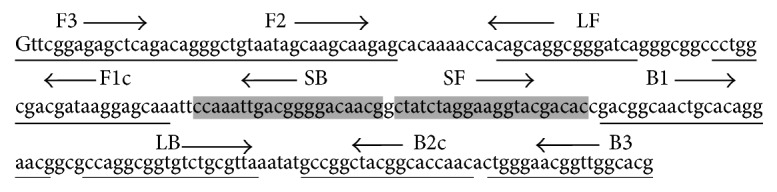
The position of LAMP and stem primers on* T. brucei gambiense*-specific glycoprotein (TgsGP) sequence section. The stem section lies between primers F1c and B1. The LAMP product is composed of the sequence between primers F2 and B2c. The outer forward and backward primers (F3/B3) displace the strand and therefore do not form part of the final LAMP product.

The LAMP and stem primers were manually designed based on the TgsGP sequence (Genbank accession number AJ27795) and following the published primer conditions [25, 28]. The primers were designed from the same sequence section as the previously published TgsGP LAMP test [24] (Figure 1). We could not insert the stem primers between the published standard LAMP primers because the stem section could not accommodate them. The primers included the outer forward and backward (F3/B3), forward and backward inner primers (FIP/BIP), loop forward and backward primers (LF/LB), and stem forward and backward primers (SF/SB) (Figure 1). All the primers were checked for specificity using the nucleotide basic local alignment search tool (BLASTn) against human DNA and other human infectious pathogens. The* T. brucei gambiense* stem LAMP test was analyzed in the following combinations: (i) with outer primers, F3/B3, and (ii) without outer primers. The standard* T. brucei gambiense* LAMP consisted of F3/B3, FIP/BIP, and LF/LB primers [24].

### 2.4. The Real-Time LAMP Reactions

To improve the sensitivity of stem LAMP test, concentration of four reaction components was optimized using Taguchi method followed by regression analysis to select concentration optima for each reagent [29]. Briefly the concentration of FIP and BIP was varied from 30 to 60 pmoles, stem forward and backward (SF/SB) primers were varied from 10 to 30 pmoles, dNTPs were varied from 1 to 3 mM, and extra magnesium was varied from 0 to 3 mM. The 1x ThermoPol reaction buffer contained 20 mM Tris-HCl (pH 8.8), 10 mM KCl, 10 mM (NH_4_)_2_SO_4_, 2 mM MgSO_4_, and 0.1% Triton X-100. The* Bst* 3.0 DNA polymerase (New England Biolabs, MA, USA) was at 0.5 *μ*L and SYTO-9 fluorescence dye was used at 3.0 *μ*M (Molecular Probes, Oregon, USA). The template was ~100 pg of purified trypanosome DNA from* T. brucei gambiense* isolate PT41. The LAMP reactions were performed at 58, 63, and 65°C for 60 minutes using the Rotorgene 6000 (Qiagen, Victoria, Australia) and data acquired on HRM channel (460–510 nm) followed by reaction inactivation at 80°C for 5 minutes. Once the test conditions for stem LAMP test were selected, the test analytical sensitivity was determined and compared with standard LAMP test using tenfold serial dilution of PT41 DNA prepared from ~100 ng (1.0 × 10^6^ trypanosomes/mL) to ~1 fg (0.01 trypanosomes/mL). All tests were done in duplicate and repeated once after two weeks. The LAMP test reaction conditions determined in the Rotorgene 6000 were duplicated using the portable and battery operated ESE Quant Tube Scanner amplification unit (Qiagen, Stockach, Germany) and used in analyses of tsetse samples. The results were obtained in real-time using the FAM channel in the scanner studio software. The template was set at 1 *μ*L for the DNA and 2 *μ*L for all other templates. The tests specificity was checked with tsetse DNA and the closely related* T. brucei rhodesiense* and* T. b. brucei* DNA.

### 2.5. Detection and Confirmation of LAMP Products

The formation of LAMP product was first monitored in real-time through fluorescence of SYTO-9 dye in Rotorgene 6000. To confirm that the stem LAMP test formats amplified the predicted target, melt curves were acquired after amplification using Rotorgene at 1°C steps, with a hold of 30 s, from 62°C to 96°C [30]. The products were later analyzed in 2.0% agarose gels stained with SYBR® safe DNA gel stain. In the portable ESE Quant unit, the products were confirmed using the melt curves acquired using the ESE melt software as described by the manufacturer.

### 2.6. LAMP Diagnostic Performance

The tests diagnostic sensitivity, specificity, accuracy, and positive and negative predictive values were done using 28 archived DNA samples that had been prepared from microscopically positive patients and vectors (positive samples) [12, 27] and 63 microscopically negative samples from HAT foci (negative samples). In addition, a total of 19 DNA samples previously prepared from tsetse midguts of nonexposed tsetse flies were used as negative controls. The samples had been stored at −80°C for over 7 years. The performance indices were number of true positives (TP), true negatives (TN), false positives (FP), and false negatives (FN). The diagnostic sensitivity (ability of the test to detect a true positive) was expressed as TP/(TP + FN) and specificity (ability of the test to exclude a true negative) as TN/(TN + FP). The positive predictive value (PPV), negative predictive value (NPV), and diagnostic accuracy were also determined. The indices were calculated using the online statistical programme https://department.obg.cuhk.edu.hk/researchsupport/SenSpc.asp.

## 3. Results

**Figure 2:**
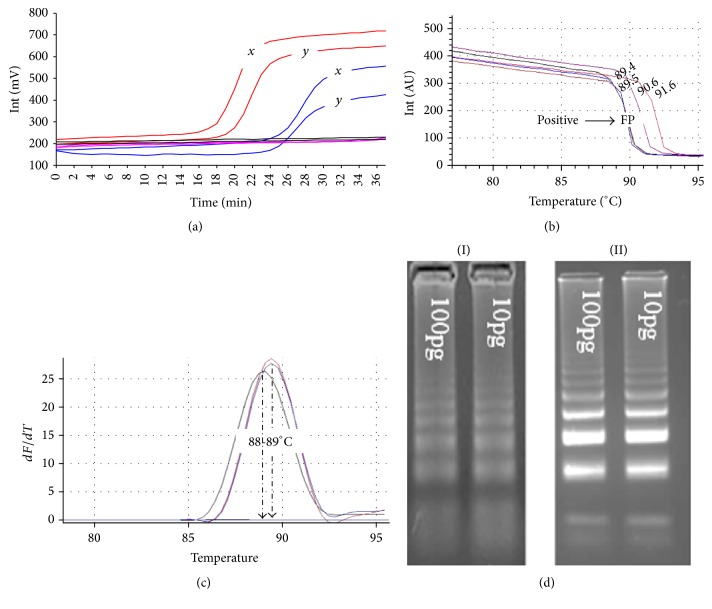
(a) The tube scanner fluorometric output of stem LAMP (red) and standard LAMP (blue) tests using ~10 pg of DNA from isolate PT41 (*x*) and DNA prepared from CSF (*y*) of a confirmed HAT patient. The unit reports the fluorescence in millivolts (mV) on *y*-axis and time in minutes on the *x*-axis. (b) The acquisition of melts curves for PT41, DNA prepared from CSF, and supernatant prepared from a tsetse fly sample spiked with B014 DNA. *T*_*m*_ was 89.5°C for PT41 and DNA from CSF patient and 89.4°C for B014 in tsetse fly sample. The nonspecific product (FP) is induced for illustration and showed *T*_*m*_ that ranged from 72 to 76°C and 90 to 92°C (c). The duplication of melts peaks for PT41, CSF, and B014 DNA samples using Rotorgene 6000. The melt peaks showed *T*_*m*_ of ~89°C for PT41 and CSF DNA and ~ 88°C for B014. (d) The amplicons for stem LAMP test (I) without outer primers and (II) with outer primers.

The Taguchi method estimated the optimum concentration for the reaction components for* T. brucei gambiense* stem LAMP as follows: FIB/BIP at 35 pmol, SF/SB 15 pmol, 2 mM dNTPs, and 1.5 mM of extra magnesium sulphate. The optimum reaction temperature was determined at 63°C and 35 minutes was chosen as the test cut-off point since the lowest amplification level was recorded at 29 minutes. There was appearance of false positives (primer-dimers) when the reactions were ran beyond 48 min and 63 minutes for stem LAMP format with outer and without outer primers, respectively. The analytical sensitivity of both stem LAMP test formats was ~10 trypanosomes/mL (~1 pg) compared to ~100 trypanosomes/mL for standard LAMP test. Furthermore, we recorded sensitivity levels of ~1 trypanosome/mL (~100 fg) for stem LAMP test formats but results were inconsistent (i.e., 3 out of 6 replicates of 1.0 × 10^−6^ dilution were positive). The stem LAMP formats indicated faster time to results and with similar product with a melting temperature of ~89°C (Table 1 and Figures 2(a)–2(c)), though the amplicons for LAMP test without outer primers were less bright (Figure 2(d)). Overall the stem LAMP test formats recorded superior diagnostic sensitivity of 71.4% compared to standard LAMP test with 57.1% and PCR with 46.4%. Moreover the LAMP test format without outer primers showed higher diagnostic accuracy of 83.5%, positive predictive value of 74.1%, and negative predictive value of 87.5% (Table 2). These superior diagnostic indices translated into higher detection of pathogen DNA of 9/16 compared to standard LAMP test with 5/16 using tsetse fly samples spiked with trypanosome DNA (Table 1).

## 4. Discussion

The stem LAMP test formats described here indicated improved diagnostic performance compared to the standard LAMP test. This is attributed to the use of two sets of reaction accelerating primers (loop and stem primers) compared to one-set primers (loop primers) in the standard LAMP format. The loop primers accelerate the reaction by priming the stable loops between FIP/BIP primers [26] while the stem primers presumably work by priming off the newly generated amplicon as it is made transiently single-stranded during DNA replication [25]. This sequential amplification mimics nested PCR format and leads to increased formation of LAMP product and hence improvement in sensitivity. The use of both stem primers concurrently and in their reverse orientation indicated the best reaction performance (Figure 1). Any other combination and orientation increased reaction time and significantly reduced test sensitivity as much as 100-fold. The stem LAMP test formats amplified identical product with the standard LAMP test as indicated through melt curves. This is expected since the final LAMP product is between primers F2 and B2c and the stem primers are within this region. More promising is the ability of the new test to indicate a higher sensitivity with supernatant as compared to extracted DNA prepared from the same sample (Table 1). It appears that more DNA is lost through kit extraction than when the sample is processed through boiling. The ability to use supernatant shortens the experimental procedure; nevertheless before supernatant can be relied upon as template for LAMP reactions, protocols for template purification and buffers that stabilise DNA in the supernatant need to be developed.

The omission of the outer primers in an optimized stem LAMP test format did not reduce the test sensitivity (Table 2) although the amplicons were less bright after electrophoresis (Figure 2(d)). Indeed the format without outer primers surprisingly showed superior diagnostic test performance compared to other LAMP test formats (Table 2). The use of four sets of primers (eight primers) in the stem LAMP format with outer primers increases the possibility of forming nonspecific products and hence the lower diagnostic accuracy of 73.6% and PPV value of 55.6%. The primary role of the outer primers is supposedly to displace the newly synthesised strands into a single strand making it available for extension by either inner primer [28]. It appears that other primers are able to displace and extend the strand in absence of outer primers albeit less efficiently. The possibility of omitting the outer primers is advantageous because it allows more flexibility of positioning both the loop and stem primers and especially in shorter sequences. We noted that the stem primers may not be appropriate for all LAMP tests. In separate studies, the inclusion of stem primers in the RIME LAMP test [12] did not alter the test sensitivity and lead to appearance of spurious products.

Since the inception of LAMP technology, the product detection formats have been developed towards visual inspection of result. Unfortunately most detection formats available are nonspecific and do not offer any step for confirming the predicted product. The real-time ESE Quant unit used in this study uses nonspecific fluorescence dye but provides a product confirmation step through acquisition of melt curves after amplification (Figure 2(b)). Melt curves can indicate different reaction products based on their shape and peaks (Figures 2(b) and 2(c)). In PCR the primer-dimers usually melt at lower temperatures since they are smaller than the desired product while the nonspecific product in LAMP can have either a lower or higher *T*_*m*_ than the desired product since more primers are used (Figure 1). Determination of nonspecific products is valuable since their presence reduces the amplification efficiency and ultimately the accuracy of the test. Ideally each LAMP test should give identical melt curves and/or peaks if the product is composed of identical sequence. Any base difference from the desired sequence will be detected as either a shift in the melt curve (Figure 2(b)) or peaks (Figure 2(c)) and hence the basis for determination of nonspecific product. The different melting temperatures observed in this study between B014 spiked samples and other positive samples may indicate a point mutation resulting in transversion of a single or more bases. Indeed transversion within TgsGP sequence has been reported in isolates from Cameroun [21] and B014 DNA was isolated from human in Fontem, Cameroon, in 1988. The reliability of the melt curves obtained here using ESE melt curve software was ascertained by duplication of similar results using Rotorgene 6000 (Figure 2(c)).

The detection of trypanosomes in the tsetse flies is routinely done through dissection followed by microscopic examination, methods that are time-consuming and more often inaccurate. This implies xenomonitoring through dissection is impractical. The stem TgsGP LAMP test reported here partly fills this gap since it is specific for* T. brucei gambiense* and shows improved diagnostic sensitivity and accuracy (Table 1) compared to the standard TgsGP LAMP test [24]. Similar LAMP test results were recorded albeit with higher sensitivity with RIME LAMP [27, 31]. Since RIME LAMP cannot differentiate human pathogenic trypanosomes from* T. b. brucei*, it is further proposed that the stem TgsGP LAMP test can be used in algorithm with RIME LAMP tests to identify* T. brucei gambiense* DNA among RIME LAMP positive samples.

## 5. Conclusions

This study provides a* T. brucei gambiense* stem LAMP test format that combines with real-time technology to detect and confirm pathogen DNA in tsetse samples. The stem LAMP test formats show improved diagnostic sensitivity and accuracy compared to the standard LAMP test. The LAMP format without outer primers indicated the best performance. The combination of LAMP test and real-time technology offers visual inspection and confirmation of results at the point of use, characteristics that are ideal for a surveillance test. Such combination can provide data on the distribution of trypanosomes and hence play a crucial role in guiding the vector control strategies since not all the vector population will carry infections.

## Figures and Tables

**Table 1:** The analysis of tsetse fly samples using *T. brucei gambiense* standard and stem LAMP test formats.

Source	Number of pools^a^	Template type	LAMP tests					
			Standard LAMP^b^	*C*_*T*_ value^c^	Stem LAMP_w_	*C*_*T*_ value	Stem LAMP_wn_	*C*_*T*_ value
Midgut	5	DNA	+	26	+	19	+	19
		Supernatant	+	23	+	15	+	15
	10	DNA	−	—	+	21	+	21
		Supernatant	+	25	+	18	+	18
	15	DNA	−	—	−	—	−	—
		Supernatant	−	—	+	24	+	24
	20	DNA	−	—	−	—	−	—
		Supernatant	−	—	−	—	−	—

Whole fly	1	DNA	+	28	+	20	+	20
		Supernatant	+	26	+	19	+	20
	5	DNA	−	—	−	—	−	—
		Supernatant	−	—	+	—	+	23
	10	DNA	−	—	−	—	−	—
		Supernatant	−	—	−	—	+	28^†^

^a^Insectary* G. pallidipes* (midguts or whole fly).

^b^Published TgsGP LAMP test [24].

^c^Cycle threshold (minutes).

^†^2 out of 6 replicates showed the predicted product.

**Table 2:** The diagnostic performance of TgsGP LAMP test formats and PCR using archived reference samples.

Indices (%)	LAMP tests			TgsGP-PCR
	Standard LAMP	Stem LAMP_w_^a^	Stem LAMP_wn_^b^	
Sensitivity	57.1	71.4	71.4	46.4
Specificity	79.4	74.6	88.9	95.2
Diagnostic accuracy	72.5	73.6	83.5	80.2
PPV	55.2	55.6	74.1	81.3
NPV	80.1	85.1	87.5	80

^a^Stem LAMP test with outer primers.

^b^Stem LAMP test without outer primers.
